# Availability, Formulation, Labeling, and Price of Low-sodium Salt Worldwide: Environmental Scan

**DOI:** 10.2196/27423

**Published:** 2021-07-14

**Authors:** Xuejun Yin, Hueiming Liu, Jacqui Webster, Kathy Trieu, Mark D Huffman, J Jaime Miranda, Matti Marklund, Jason H Y Wu, Laura K Cobb, Ka Chun Li, Sallie-Anne Pearson, Bruce Neal, Maoyi Tian

**Affiliations:** 1 The George Institute for Global Health University of New South Wales Sydney Australia; 2 Sydney Institute for Women, Children and their Families Sydney Local Health District Sydney Australia; 3 Feinberg School of Medicine Northwestern University Evanston, IL United States; 4 CRONICAS Centre of Excellence in Chronic Diseases Universidad Peruana Cayetano Heredia Lima Peru; 5 Department of Medicine School of Medicine Universidad Peruana Cayetano Heredia Lima Peru; 6 Department of Epidemiology Johns Hopkins Bloomberg School of Public Health Baltimore, MD United States; 7 Department of Public Health and Caring Sciences Uppsala University Uppsala Sweden; 8 Resolve to Save Lives Initiative of Vital Strategies New York City, NY United States; 9 Centre for Big Data Research in Health University of New South Wales Sydney Australia; 10 School of Public Health Imperial College London London United Kingdom; 11 The George Institute for Global Health at Peking University Health Science Center Beijing China

**Keywords:** low-sodium salt, salt substitute, availability, formulation, labeling, price, sodium, salt, blood pressure, cardiology

## Abstract

**Background:**

Regular salt is about 100% sodium chloride. Low-sodium salts have reduced sodium chloride content, most commonly through substitution with potassium chloride. Low-sodium salts have a potential role in reducing the population's sodium intake levels and blood pressure, but their availability in the global market is unknown.

**Objective:**

The aim of this study is to assess the availability, formulation, labeling, and price of low-sodium salts currently available to consumers worldwide.

**Methods:**

Low-sodium salts were identified through a systematic literature review, Google search, online shopping site searches, and inquiry of key informants. The keywords “salt substitute,” “low-sodium salt,” “potassium salt,” “mineral salt,” and “sodium reduced salt” in six official languages of the United Nations were used for the search. Information about the brand, formula, labeling, and price was extracted and analyzed.

**Results:**

A total of 87 low-sodium salts were available in 47 out of 195 (24%) countries worldwide, including 28 high-income countries, 13 upper-middle-income countries, and 6 lower-middle-income countries. The proportion of sodium chloride varied from 0% (sodium-free) to 88% (as percent of weight; regular salt is 100% sodium chloride). Potassium chloride was the most frequent component with levels ranging from 0% to 100% (potassium chloride salt). A total of 43 (49%) low-sodium salts had labels with the potential health risks, and 33 (38%) had labels with the potential health benefits. The median price of low-sodium salts in high-income, upper-middle-income, and lower-middle-income countries was US $15.00/kg (IQR 6.4-22.5), US $2.70/kg (IQR 1.7-5.5), and US $2.90/kg (IQR 0.50-22.2), respectively. The price of low-sodium salts was between 1.1 and 14.6 times that of regular salts.

**Conclusions:**

Low-sodium salts are not widely available and are commonly more expensive than regular salts. Policies that promote the availability, affordability, and labeling of low-sodium salts should increase uptake, helping populations reduce blood pressure and prevent cardiovascular diseases.

**International Registered Report Identifier (IRRID):**

RR2-10.1111/jch.14054

## Introduction

Cardiovascular diseases (CVDs) are the leading causes of death worldwide, and high blood pressure is a leading risk factor for CVDs [1,2]. Dietary sodium intake is a strong causal determinant of blood pressure levels [3,4]. The World Health Organization (WHO) recommends reducing sodium intake as one of the *best-buy* strategies for lowering the population-level risk of CVDs and set a goal of 30% reduction in the population [5]. Different strategies are proposed to reduce dietary sodium intake. Although the effect of sodium reduction interventions on blood pressure has been demonstrated in controlled research settings, there are few examples of sodium reduction at a population scale [6,7]. Innovative strategies are needed to reduce sodium intake from processed foods, food eaten outside the home, and home cooked foods.

Regular salt is about 100% sodium chloride. Low-sodium salt has reduced sodium chloride content, most commonly through substitution with potassium chloride or magnesium sulphate. There is a growing body of evidence supporting the use of low-sodium salts as an effective intervention to reduce dietary sodium intake, lower blood pressure, and thereby prevent the adverse consequences of high blood pressure [8,9]. Although one concern with low-sodium salts enriched with potassium was the potential risk for people who have advanced chronic kidney disease (CKD) due to hyperkalemia, a comparative risk assessment model estimated that nationwide replacement of the salt supply with potassium-enriched, low-sodium salt in China could result in the prevention of 1 in every 9 deaths from CVD and a net benefit with the use of low-sodium salts even in individuals with CKD [10]. The approach of integrating low-sodium salts as a public health intervention can potentially reduce dietary sodium intake at the population level through reformulating manufactured foods with low-sodium salts, replacing cooking salt used at home or in restaurants with low-sodium salts, or a combination thereof. Thus, population uptake of low-sodium salts has been recommended as one of seven priority strategies to reduce population sodium consumption [11].

Low-sodium salts may have a potential role in enhancing equitable access to effective CVD prevention worldwide as long as it is affordable and has sufficient reach to countries regardless of the income levels. However, little is known about the availability and accessibility of this emerging product in the global market or about factors that may affect equitable uptake including formulation, price, or labeling. We, therefore, performed a systematic search of low-sodium salts to understand better their availability, formulation, labeling, and the price in different countries.

## Methods

This study was a systematic search of low-sodium salts conducted from October 2019 to September 2020. The study protocol has been published previously [12].

### Definition of Low-sodium Salt

Low-sodium salts were defined as table salt or cooking salt that replaced sodium chloride content with other minerals such as potassium chloride or magnesium sulphate. The sodium content and terminology for low-sodium salts can vary. For example, in some cases, the term *salt substitute* is used as a synonym for low-sodium salt. In this study, we use the term *low-sodium salts* as a category, including both sodium-reduced and sodium-free salts. All low-sodium salts available for retail purchase were eligible for inclusion, but those that had ceased production were excluded.

### Search Strategy

Low-sodium salts were identified from four sources. First, a systematic literature search was conducted in MEDLINE, Embase, and Cochrane Library from inception through March 2020 without language restrictions. The search strategies are listed in Multimedia Appendix 1 and include the keywords of “salt substitute,” “low sodium salt,” “potassium salt,” “mineral salt,” and “sodium reduced salt.” Second, we searched major global online shopping sites, including Amazon, eBay, Walmart, JD, and RedMart, to identify low-sodium salts. Third, we executed a search using Google advanced engine with a Google Chrome browser from Australia. Initial keywords included different terms describing low-sodium salts. The search strategies are shown in Textbox 1. Initial keywords and country names were combined for a Google search to identify the availability of low-sodium salts in different countries. The first 25 results of each search were examined for eligibility. This was primarily because the search results after the first 25 were generally unrelated to low-sodium salts. Six official languages of the United Nations (Arabic, Chinese, English, French, Russian, and Spanish) were used in the Google searches and online shopping website searches considering that the language would influence the ranking of resulting pages. Google searches were conducted using a Google Chrome browser. The translation from other languages to English was performed by the built-in translation service within the Google Chrome browser [13]. Fourth, we conducted semistructured interviews with key informants who have been involved in the research, manufacturing, implementation, and promotion of low-sodium salts. We used purposive and snowball sampling to recruit key informants. Academic representatives were identified through the systematic review, which was part of the broader environmental scan. Corresponding authors of eligible studies that were identified from the systematic review were contacted via corresponding authors’ email addresses. Academic representatives who participated in the interview were asked to refer the study to potential key informants from relevant government agencies or the salt manufacturing industry through a snowball sampling strategy. A total of 18 key informants from 9 countries representing all WHO regions provided information about the low-sodium salts they knew. Results from four data sources were combined, and duplicates were then removed.

**Keywords and terms in Google search strategy.**

**Initial keywords**

- “low-sodium salt,” “salt substitute,” “potassium salt,” “mineral salt,” “sodium reduced salt”

**Terms of different aspects relevant to the salt substitutes**

- Formula: “formula,” “composition,” “ingredient”
- Price: “price,” “cost”
- Country: for example, “Finland,” “China,” “Italy”
- Safety issue: “safety,” “adverse events,” “warning,” “danger,” “threatening”

To identify the low-sodium salts used in research studies, we had broad inclusion criteria on the study population and study types. All titles and abstracts of records identified in the databases were screened for eligibility. Duplicated studies were excluded. Full texts of papers after eligibility screening were obtained for independent review by two authors (XY and KCL). Differences between reviewers were resolved through discussion or consultation with a third senior investigator (MT) when necessary. Information about the low-sodium salts was extracted. Low-sodium salts were excluded if they were manufactured exclusively for a study but not available for a general population or ceased production and were no longer available in the market.

### Data Extraction and Analysis

Data were extracted by one researcher (XY) to a predesigned Microsoft Excel (Microsoft Corporation) sheet, including data source, search date, product website, brand, formulation, nutrition facts, safety warnings, and price. The price of regular salt in the same brand was also extracted from online shopping websites for price comparison. Information on the low-sodium salt was summarized by country. Price was converted into US dollars per kilogram (US $/kg) using the exchange rate at the time of the search. The international dollar of low-sodium salts by the exchange rate in the year of 2020 was also reported for considering purchasing power parities and the commodity prices [14]. The average price of low-sodium salts was compared by country income levels. The income levels were divided according to the World Bank classification [15]. Statistical analyses were performed using Microsoft Excel and SPSS (IBM Corp, Version 26).

## Results

### Summary of Identified Low-sodium Salts

**Figure 1:**
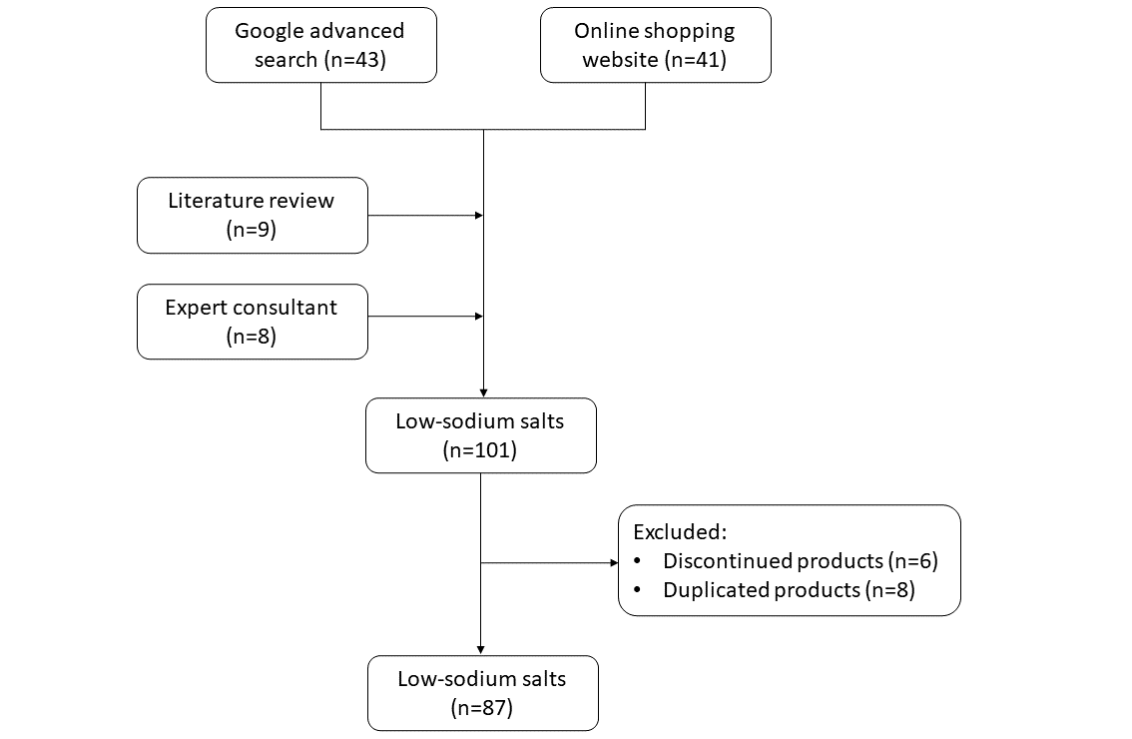
Flowchart of low-sodium salt indentification.

Our final analyses comprised 87 low-sodium salts that were identified across 47 countries. Google searches identified 43 products. Another 41 products were identified from online shopping sites. From the published research studies, 4440 abstracts were screened, 67 of them were related to low-sodium salts, and 9 brands of low-sodium salts were identified from full-text papers. A total of 8 low-sodium salts were complemented from consulting experts. There were 8 duplicated products and 6 discontinued products, which were removed from the final analysis (Figure 1). Key characteristics of all included low-sodium salts are described in Multimedia Appendix 2.

### Availability of Low-sodium Salts

**Figure 2:**
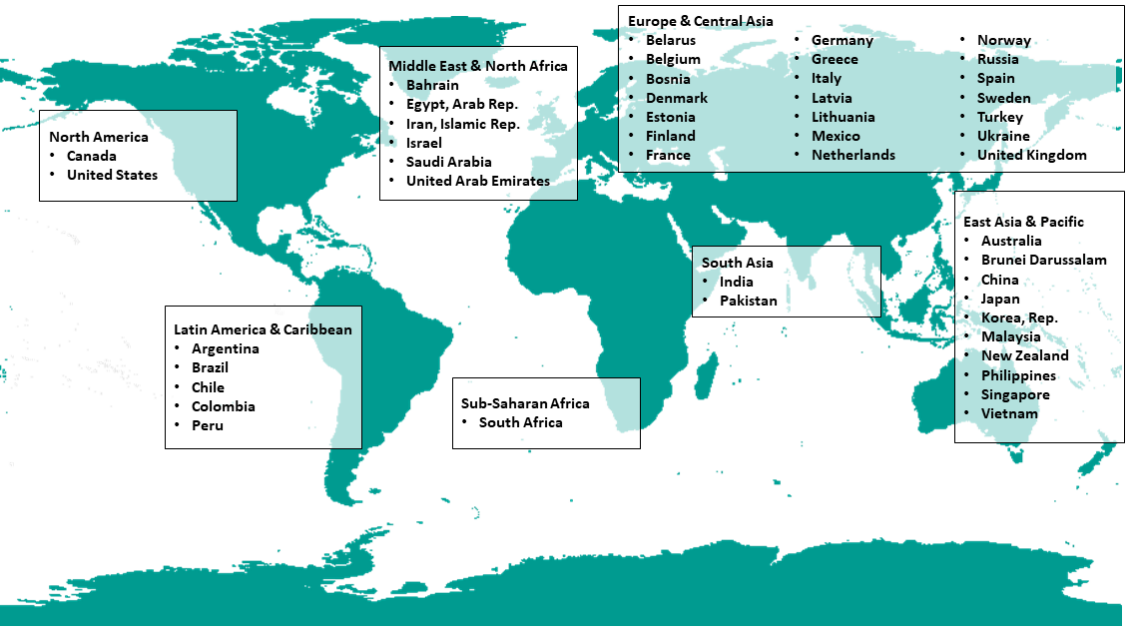
Availability of low-sodium salt in different countries.

Low-sodium salts were available in 47 countries, including 28 high-income countries, 13 upper-middle-income countries, and 6 lower-middle-income countries. The United States, Canada, the United Kingdom, Italy, China, India, France, Sweden, Argentina, and Russia produced more than 1 brand of low-sodium salt. Low-sodium salts were found in all seven world regions defined by the World Bank. Most countries with low-sodium salts were located in the Europe and Central Asia countries (n=21) and East Asia and Pacific countries (n=10; Figure 2).

### Formulation and Labeling

**Figure 3:**
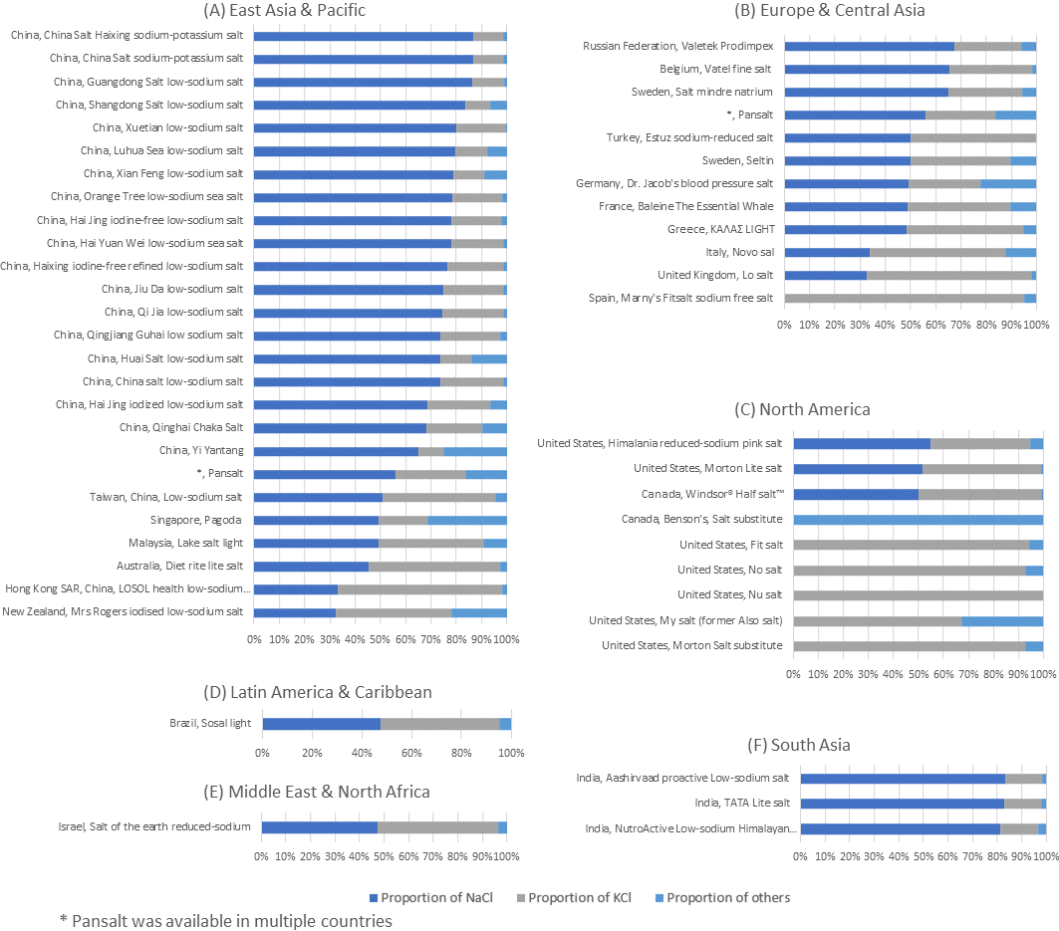
The proportions of sodium chloride (NaCl), potassium chloride (KCl), and other minerals for low-sodium salts by regions (N=51).

Among low-sodium salts, the sodium chloride varied from 0% (sodium-free) to 88% (as percent of weight, regular salt is 100% sodium chloride). Potassium chloride varied from 0% to 100%. A total of 51 low-sodium salts reported both sodium and potassium levels whose composition are shown in Figure 3. The composition of low-sodium salts varied by different countries. For example, less than 20% of the sodium chloride was replaced in low-sodium salts produced in India, while at least 50% of the sodium chloride was replaced in countries in North America, the Middle East, and Latin America. Sodium-free salt substitutes were mostly available in the United State and Canada (n=7).

Among the 87 products, 41 (46%) were iodized, 46 (53%) had nutrition facts information on their labels, and 55 (63%) had a front of package labeling indicating less sodium in low-sodium salt than regular salt. A total of 43 (49%) products had labels advising potential health risks, 33 (38%) had labels advising potential health benefits, 16 (18%) had labels with both, and 27 (31%) had labels with neither. The labels advising potential health risk was signed to warn high-risk populations who might potentially be harmed by excessive potassium intake, but the contents of these advisory labels varied. Examples of advisory warning labels on low-sodium salt packages are shown in Textbox 2. The labels advising potential health benefits included lowering sodium intake without compromising taste, being additional sources of potassium, and lowering blood pressure. Examples of health benefit labels on low-sodium salt packages are shown in Textbox 3.

**Examples of advisory warning labels found on low-sodium salt packages.**

- “A good source of potassium. Should not be used by persons on a sodium or potassium restricted diet unless approved by a physician.” (United States, Morton and Canada, Windsor)
- “For normal healthy people. Persons having diabetes, heart or kidney disease, or receiving medical treatment should consult a physician before using a salt alternative or substitute.” (United States, Nu-salt)
- “It should be used with caution among people who are not suitable for high potassium intakes, such as high-temperature workers, heavy-labour workers, renal dysfunction and hypertension patients taking antihypertensive drugs.” (China, China Salt)
- “It is recommended to use the product under medical supervision.” (Italy, Novo sal)
- “Medical advice should be sought for diet requiring low or restricted sodium or potassium intake. Not suitable for use with a certain diuretic.” (Finland, Pansalt)
- “In case of kidney or heart failure or high blood pressure, you should consult your doctor before use.” (Russia, Mediterra salt)

**Examples of health benefit labels found on low-sodium salt packages.**

- “Pansalt is a clinically-proven low-sodium salt with added essential minerals for a healthy heart. The low sodium mineral salt is the result of many years of intensive scientific research and is ideal for consumers who want to follow a healthier diet without compromising on good taste. Apart from the overall health benefits. Pansalt delivers the true taste of salt but without the harmful effects resulting in a high consumer approval rate.” (Finland, Pansalt)
- “TATA salt lite has been specially formulated to provide 15% lower sodium than ordinary salt. It is generally accepted that lower sodium in diets may assist in management of high blood pressure.” (India, TATA)
- “It is being a good source of potassium; helps you maintain normal blood pressure.” (United Kingdom, Lo salt)
- “Good for your health – potassium and a reduced consumption of sodium contribute to the maintenance of normal blood pressure. Potassium further contributes to the normal functioning of the nervous system and muscles.” (Netherlands, Nezo Light Salt)
- “Replace part of sodium chloride with potassium chloride. The taste is like regular salt. It can help reduce the intake of sodium and increase potassium intake.” (China, Yi Yan Tang)
- “It can significantly reduce excessive sodium intake and increase potassium and magnesium intake. Potassium is necessary to maintain the work of the heart, nervous system and muscle system, clear excess fluid from the body, and maintain normal blood pressure” (Russia, Valetek Prodimpex)

### Price of Low-sodium Salts

**Figure 4:**
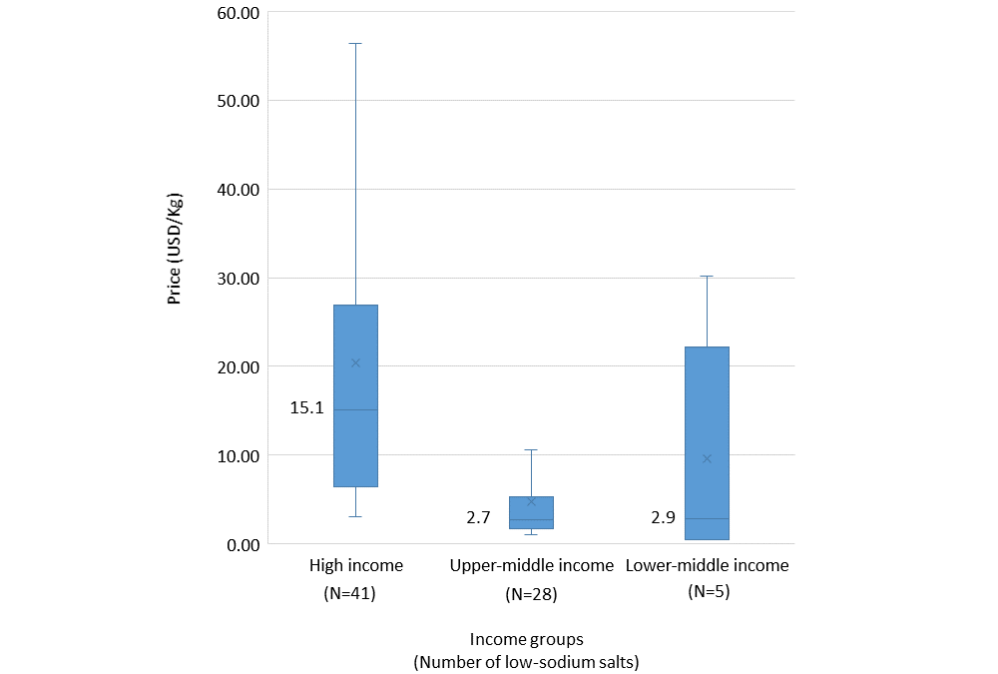
Price for low-sodium salts across country income groups.

**Figure 5:**
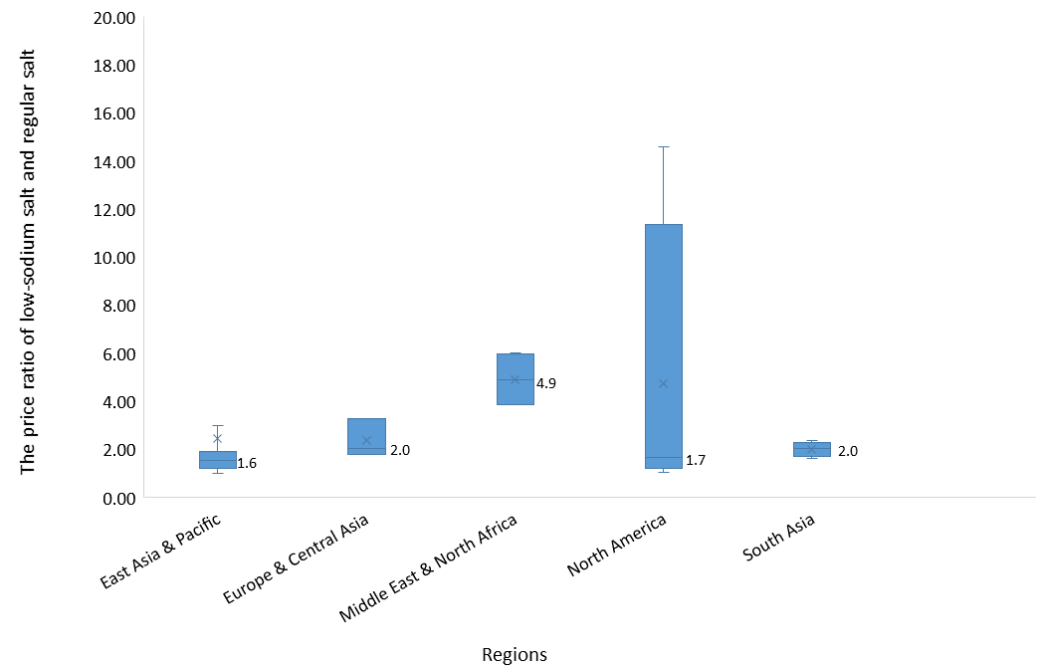
Price differential among salt manufacturers producing both low-sodium salts and regular salts by different regions.

The price of low-sodium salts varied from US $0.46/kg to US $87.00/kg. The median price of low-sodium salts in high-income, upper-middle-income, and lower-middle-income countries was US $15.10/kg (IQR 6.4-26.9), US $2.70/kg (IQR 1.7-5.3), and US $2.90/kg (IQR 0.5-22.2), respectively (Figure 4). After converting to the international dollar, the median international dollar of low-sodium salts in high-income and upper-middle-income was US $14.80/kg (IQR 8.5-32.9) and US $11.60/kg (IQR 7.4-18.4), respectively. The median international dollar of low-sodium salts in lower-middle-income countries surged to US $111.10/kg (IQR 11.0-469.9). Among salt manufacturers producing both low-sodium salts and regular salts (N=38), the price of low-sodium salts was 1.7 times the price of the regular salts. The price difference between low-sodium salt and regular salt by regions is presented in Figure 5.

## Discussion

The findings of this study indicated that low-sodium salts were only available in 24% of the countries worldwide. Noticeably, 60% of these countries were high-income countries. Prices varied between US $0.46/kg and US $87.00/kg but were consistently higher than regular salts. Sodium chloride content ranged from 0% to 88%. Advisory labels on benefits and harms of using low-sodium salts were not standardized.

The availability of low-sodium salts was limited in low- and middle-income countries. Many low- and middle-income countries are facing disproportionately large burdens of CVD due to unhealthy diet [2,16]. However, there are few effective and affordable strategies to combat the increasing burden in resource-constrained settings. Salt reduction was listed as a *best-buy* intervention by the WHO, as it is an effective intervention in low- and middle-income countries where the primary source of sodium is from salt added in cooking and food preparation, and the proportion of discretionary salt to total sodium intake is higher [17,18]. A low-sodium salt population intervention could potentially enhance equity of access to effective CVD prevention. Important next steps include the increase in production and market demand of low-sodium salts. One example of this was in 2010, the Beijing city government strengthened the supply chain to ensure that low-sodium salts were available in 28 different supermarket chains as part of a citywide salt reduction initiative [19]. Low-sodium salt is a simple and low-cost intervention, which makes it a compelling proposal for low- and middle-income countries. In high-income countries, where CVD is also a major burden and low-sodium salts are generally easy to access, low-sodium salts can serve as a complementary strategy for product reformulation and an ideal replacement added during home cooking or at the table. The approach to integrating low-sodium salts in public health interventions may vary by country depending on the major dietary source of sodium. Feasibility studies in countries with different diet patterns are needed to understand better how to introduce low-sodium salts onto the market and how to promote low-sodium salt use in different contexts.

Low-sodium salts have higher prices than regular salts in all countries where low-sodium salts are commercially available. The price of food is a vital factor that influences consumers’ behavior [20]. One cluster randomized control trial revealed that even with the same health education program, the adoption of low-sodium salts was higher in villages where the price of low-sodium salts was subsidized compared with villages without a price subsidy [21]. Results of this study showed that the prices of low-sodium salts were 1.0 to 14.6 times higher than the price of regular salts. Price differences were highest in North America, likely because low-sodium salts produced in this area had a larger proportion of potassium chloride (potassium costs about four times more than sodium) [22]. Some countries, like India, do not have readily available sources of potassium, which may further increase costs of potassium supplementation [23]. Narrowing the price difference between low-sodium salts and regular salts would promote low-sodium salt use, which can motivate manufacturers to improve low-sodium salt availability. Government subsidies and regulation can increase the affordability and reduce the price differences between low-sodium salts and regular salts, especially among individuals with limited resources who may face a disproportionate risk and burden of CVD. The low-sodium salt intervention ought to be implemented by reformulation or policies without passing the costs onto individuals who will be less likely to afford these higher prices. One real-world example of subsidies was in Beijing, where in addition to improving the supply chain, an additional 75g of low-sodium salt was provided in the 400g package [19]. Further quantitative market surveys and qualitative studies can be designed to understand the context and mechanism of the price of low-sodium salts in the future.

Low-sodium salts varied in composition. Overall, the sodium chloride was mostly replaced by potassium chloride. The acceptability of low-sodium salt taste depends on individual preferences (palate) and concentration of potassium [24]. There have been reports that unpleasant off-tastes associated with potassium chloride may prevent customers from choosing low-sodium salts [25]. However, several studies found low-sodium salts with about 30% of sodium chloride replaced by potassium chloride had a similar flavor to regular salts when used in cooking or processed food manufacturing [24,26,27]. Reducing sodium and increasing potassium in the salt used for cooking and processed food reformulation could make the salt less *salty* in taste, leading consumers to use more low-sodium salts. Effective taste-improving agents can be introduced to overcome potential sensory drawbacks, such as taste masking or umami ingredients [28]. The acceptable range of preferences for compositions of low-sodium salts used in cooking by the general population in different culture context would need to be confirmed in future research. In addition to taste acceptability, further studies regarding the benefits and risks of low-sodium salts with different sodium and potassium proportion are still necessary.

Food labeling was designed to provide information and help consumers make healthier food choices. The effect of low-sodium salts on reducing sodium intake and blood pressure has been proven in clinical trials [8,9]. The modeling study showed that the net benefits of a national implementation of low-sodium salts were substantial in preventing CVDs, and the net benefit still presented for individuals with CKD [10]. Only 33 brands of low-sodium salts were identified as having health benefit labels. Evidence-based benefits of low-sodium salt should be reported on the package to encourage people to make healthier choices in salt. Despite limited data on the dose-response relationship between the use of salt substitutes and serum potassium levels, one concern with scaling up low-sodium salts is the potential to increase the risk of hyperkalemia and sudden cardiac arrest for a small number of people who are advised to limit dietary potassium due to impaired renal function and potassium excretion [29]. Although there is little research investigating the relative benefits and risks of salt substitute use in such populations, people who have advanced CKD or who take medications that interfere with the renin-angiotensin-aldosterone axis, including potassium-sparing diuretics, may be suboptimal candidates for low-sodium salts that are enriched with potassium. Precautionary labeling on the potassium level and health warning information are important in response to these concerns, but the optimal threshold and format of such labeling are uncertain. For labels on low-sodium salts to be useful, they must be both credible and accurate. There were 43 low-sodium salts with labels advising potential risks, but 27 of them did not mention the health benefits. An overcautious label not based on scientific evidence or labels only focused on health risks might discourage people who would receive health benefits by using the low-sodium salts. The knowledge and awareness of low-sodium salts is generally low in recent baseline surveys done for the trials with low-sodium salts in China and India [30,31]. A survey conducted in India to assess the knowledge and awareness of low-sodium salts among doctors indicated 71.5% of participants did not know about their contraindications in patients with severe renal disease, cardiac problems, and patients on potassium‑sparing diuretics [32]. Adequate education campaigns together with regulations on the labeling are imperative to ensure that regulators, the salt industry, food scientists, clinicians, and consumers are aware of the level of potassium additives and how to read the labeling information appropriately.

One strength of this study lies in the careful searches across diverse data sources to describe the landscape of low-sodium salts. We complemented conventional systematic review methods with online searches and key informant consultation to identify as many products as possible. However, results from Google searches are dynamic and can be difficult to replicate, and thus, such a method can only reflect the landscape for a short period. The changes in availability, formulation, labeling, and prices were outside the scope of this study. Additionally, only low-sodium salts having an online presence were accessed in the study. The availability of low-sodium salts may be underestimated in low- and middle-income countries due to the challenges of access to available online information in these settings. Keywords in all six major languages of the United Nations were used to maximize the possibility of identifying low-sodium salts in non-English speaking countries. However, this may still miss many countries. We tried to minimize the risk of omitting low-sodium salts by using complementary information sources, including systematic reviews and consulting key informants from a diverse set of countries based on income categories.

This study’s results provide evidence on the availability, formulation, labeling, and price of low-sodium salts worldwide. These results can be used to inform efforts to scale up low-sodium salt use as an effective public health intervention in different countries. We therefore recommend making low-sodium salts more widely available, providing accurate and standardized labeling, and subsidizing the price of low-sodium salt. Future studies on how to promote the production, sale, adoption, spread, scale-up, and sustainability of low-sodium salts in diverse settings will also help governments take the necessary steps to reduce population-level dietary sodium consumption through the widespread use of low-sodium salt.

## Appendix

Multimedia Appendix 1 Database search strategy.

Multimedia Appendix 2 Key characteristics of all included low-sodium salts.

## Abbreviations

CKD: chronic kidney disease
CVD: cardiovascular disease
WHO: World Health Organization

## References

[1] GBD 2017 Causes of Death Collaborators (2018). Global, regional, and national age-sex-specific mortality for 282 causes of death in 195 countries and territories, 1980-2017: a systematic analysis for the Global Burden of Disease Study 2017. Lancet.

[2] GBD 2017 Diet Collaborators (2019). Health effects of dietary risks in 195 countries, 1990-2017: a systematic analysis for the Global Burden of Disease Study 2017. Lancet.

[3] Huang L, Trieu K, Yoshimura S, Neal B, Woodward M, Campbell NRC, Li Q, Lackland DT, Leung AA, Anderson CAM, MacGregor GA, He FJ (2020). Effect of dose and duration of reduction in dietary sodium on blood pressure levels: systematic review and meta-analysis of randomised trials. BMJ.

[4] Forouhi NG, Unwin N (2019). Global diet and health: old questions, fresh evidence, and new horizons. Lancet.

[5] (2013). A global brief on hypertension: silent killer, global public health crisis: World Health Day 2013. World Health Organization.

[6] Barberio AM, Sumar N, Trieu K, Lorenzetti DL, Tarasuk V, Webster J, Campbell NRC, McLaren L (2017). Population-level interventions in government jurisdictions for dietary sodium reduction: a Cochrane Review. Int J Epidemiol.

[7] Ponce-Lucero V, Saavedra-Garcia L, Cateriano-Arévalo E, Perez-Leon S, Villarreal-Zegarra D, Horna-Alva D, Miranda JJ (2020). Parents' perceptions about salt consumption in urban areas of Peru: formative research for a social marketing strategy. Nutrients.

[8] Peng Y, Li W, Wen X, Li Y, Hu J, Zhao L (2014). Effects of salt substitutes on blood pressure: a meta-analysis of randomized controlled trials. Am J Clin Nutr.

[9] Hernandez AV, Emonds EE, Chen BA, Zavala-Loayza AJ, Thota P, Pasupuleti V, Roman YM, Bernabe-Ortiz A, Miranda JJ (2019). Effect of low-sodium salt substitutes on blood pressure, detected hypertension, stroke and mortality. Heart.

[10] Marklund M, Singh G, Greer R, Cudhea F, Matsushita K, Micha R, Brady T, Zhao D, Huang L, Tian M, Cobb L, Neal B, Appel LJ, Mozaffarian D, Wu JHY (2020). Estimated population wide benefits and risks in China of lowering sodium through potassium enriched salt substitution: modelling study. BMJ.

[11] Ide N, Ajenikoko A, Steele L, Cohn J, J Curtis C, Frieden TR, Cobb LK (2020). Priority actions to advance population sodium reduction. Nutrients.

[12] Yin X, Liu H, Trieu K, Webster J, Farrand C, Li K, Pearson S, Tian M (2020). The effectiveness, feasibility, and acceptability of low-sodium salts worldwide: an environmental scan protocol. J Clin Hypertens (Greenwich).

[13] Google Help.

[14] (2020). Implied PPP conversion rate. International Monetary Fund.

[15] World Bank country and lending groups. World Bank Data Help Desk.

[16] Gheorghe A, Griffiths U, Murphy A, Legido-Quigley H, Lamptey P, Perel P (2018). The economic burden of cardiovascular disease and hypertension in low- and middle-income countries: a systematic review. BMC Public Health.

[17] Thout SR, Yu J, Tian M, Huffman MD, Arnott C, Li Q, Devarsetty P, Johnson C, Pettigrew S, Neal B, Wu JHY (2020). Rationale, design, and baseline characteristics of the Salt Substitute in India Study (SSiIS): the protocol for a double-blinded, randomized-controlled trial. J Clin Hypertens (Greenwich).

[18] Bhat S, Marklund M, Henry ME, Appel LJ, Croft KD, Neal B, Wu JHY (2020). A systematic review of the sources of dietary salt around the world. Adv Nutr.

[19] Shao S, Hua Y, Yang Y, Liu X, Fan J, Zhang A, Xiang J, Li M, Yan LL (2017). Salt reduction in China: a state-of-the-art review. Risk Manag Healthc Policy.

[20] Wolfson JA, Ramsing R, Richardson CR, Palmer A (2019). Barriers to healthy food access: associations with household income and cooking behavior. Prev Med Rep.

[21] Li N, Yan LL, Niu W, Yao C, Feng X, Zhang J, Shi J, Zhang Y, Zhang R, Hao Z, Chu H, Zhang J, Li X, Pan J, Li Z, Sun J, Zhou B, Zhao Y, Yu Y, Engelgau M, Labarthe D, Ma J, MacMahon S, Elliott P, Wu Y, Neal B (2016). The effects of a community-based sodium reduction program in rural China - a cluster-randomized trial. PLoS One.

[22] Drewnowski A, Rehm C, Maillot M, Monsivais P (2015). The relation of potassium and sodium intakes to diet cost among U.S. adults. J Hum Hypertens.

[23] Saha M, Maurya BR, Bahadur I, Kumar A, Meena VS, Meena VS, Maurya BR, Verma JP, Meena RS (2016). Can potassium-solubilising bacteria mitigate the potassium problems in India?. Potassium Solubilizing Microorganisms for Sustainable Agriculture.

[24] Saavedra-Garcia L, Bernabe-Ortiz A, Gilman R, Diez-Canseco F, Cárdenas MK, Sacksteder K, Miranda JJ (2015). Applying the triangle taste test to assess differences between low sodium salts and common salt: evidence from Peru. PLoS One.

[25] Sinopoli DA, Lawless HT (2012). Taste properties of potassium chloride alone and in mixtures with sodium chloride using a check-all-that-apply method. J Food Sci.

[26] Li N, Prescott J, Wu Y, Barzi F, Yu X, Zhao L, Neal B, China Salt Substitute Study Collaborative Group (2009). The effects of a reduced-sodium, high-potassium salt substitute on food taste and acceptability in rural northern China. Br J Nutr.

[27] Braschi A, Gill L, Naismith DJ (2009). Partial substitution of sodium with potassium in white bread: feasibility and bioavailability. Int J Food Sci Nutr.

[28] Cepanec Katica, Vugrinec Sašenka, Cvetković Tanja, Ranilović Jasmina (2017). Potassium Chloride-Based Salt Substitutes: A Critical Review with a Focus on the Patent Literature. Compr Rev Food Sci Food Saf.

[29] Kovesdy CP, Matsushita K, Sang Y, Brunskill NJ, Carrero JJ, Chodick G, Hasegawa T, Heerspink HL, Hirayama A, Landman GWD, Levin A, Nitsch D, Wheeler DC, Coresh J, Hallan SI, Shalev V, Grams ME, Prognosis Consortium CKD (2018). Serum potassium and adverse outcomes across the range of kidney function: a CKD Prognosis Consortium meta-analysis. Eur Heart J.

[30] Neal B, Tian M, Li N, Elliott P, Yan L, Labarthe D, Huang L, Yin X, Hao Z, Stepien S, Shi J, Feng X, Zhang J, Zhang Y, Zhang R, Wu Y (2017). Rationale, design, and baseline characteristics of the Salt Substitute and Stroke Study (SSaSS)-A large-scale cluster randomized controlled trial. Am Heart J.

[31] Yu J, Thout S, Li Q, Tian M, Marklund M, Arnott C, Huffman MD, Praveen D, Johnson C, Huang L, Pettigrew S, Neal B, Wu JHY (2021). Effects of a reduced-sodium added-potassium salt substitute on blood pressure in rural Indian hypertensive patients: a randomized, double-blind, controlled trial. Am J Clin Nutr.

[32] Fathima K, Bhargava M (2018). Salt reduction and low-sodium salt substitutes: awareness among health-care providers in Mangalore, Karnataka. Indian J Community Med.

