# Supplementary material for: Availability, Formulation, Labeling, and Price of Low-sodium Salt Worldwide: Environmental Scan
Source: JMIR Public Health Surveill. 2021 Jul 14;7(7):e27423. doi: 10.2196/27423 (PMC8319774; doi:10.2196/27423)
Supplement: Multimedia Appendix 1 [file publichealth_v7i7e27423_app1.docx]

Database: Ovid MEDLINE(R) and Embase 1946 to present, Cochrane library

1. Salt.ti,ab
2. salt substitut$.ab,ti.
3. (low sodium adj5 salt).ti,ab.
4. (sodium free adj5 salt).ti,ab.
5. salt replac$.ti,ab.
6. mineral adj5 salt.ti,ab.
7. smart salt.ti,ab.
8. sodium reduced adj5 salt.ti,ab.
9. health$ salt.ti,ab.
10. potassium adj5 salt.ti,ab.
11. potassium-rich* adj5 salt.ti,ab.
12. lite salt.ti,ab.
13. sodium adj5 potassium.ti,ab.
14. NaCl.ti,ab.
15. 1 and 13
16. 1 and 14
17. or/2-12,15,16
18. limit 17 to humans
